# Navigational Bronchoscopy versus Transthoracic Biopsy for Lung Nodules

**DOI:** 10.1056/NEJMoa2414059

**Published:** 2025-05-18

**Authors:** Robert J. Lentz, Katherine Frederick-Dyer, Virginia B. Planz, Tatsuki Koyama, Matthew C. Aboudara, Sameer K. Avasarala, Jonathan D. Casey, George Z. Cheng, Pierre-François D’Haese, Jennifer D. Duke, Eric L. Grogan, Todd C. Hoopman, Joyce Johnson, James M. Katsis, Jonathan S. Kurman, See-Wei Low, Kamran Mahmood, Otis B. Rickman, Lance Roller, Cristina Salmon, Samira Shojaee, Briana Swanner, Momen M. Wahidi, Charla Walston, Gerard A. Silvestri, Lonny Yarmus, Najib M. Rahman, Fabien Maldonado

**Affiliations:** 1 Division of Allergy, Pulmonary, and Critical Care Medicine, Vanderbilt University Medical Center, Nashville, TN; 2 Department of Thoracic Surgery, Vanderbilt University Medical Center, Nashville, TN; 3 Department of Veterans Affairs Medical Center, Nashville, TN; 4 Department of Radiology and Radiological Sciences, Vanderbilt University Medical Center, Nashville, TN; 5 Department of Biostatistics, Vanderbilt University Medical Center, Nashville, TN; 6 Division of Pulmonary and Critical Care Medicine, St. Luke's Health System, University of Missouri at Kansas City, Kansas City, MO; 7 Division of Pulmonary, Critical Care, and Sleep Medicine, University Hospitals, Case Western Reserve University School of Medicine, Cleveland, OH; 8 Trial Innovation Center and Center for Learning Healthcare, Vanderbilt Institute for Clinical and Translational Research, Nashville, TN; 9 Division of Pulmonary, Critical Care, and Sleep Medicine, University of California San Diego, La Jolla, CA; 10 Vanderbilt University School of Engineering, Nashville, TN; 11 Pulmonary and Critical Care, Kootenai Health, Coeur d’Alene, ID; 12 Department of Pathology, Vanderbilt University Medical Center, Nashville, TN; 13 Division of Pulmonary and Critical Care Medicine, Rush University Medical Center, Chicago, IL; 14 Division of Pulmonary, Critical Care and Sleep Medicine, Medical College of Wisconsin, Milwaukee, WI; 15 Division of Pulmonary Medicine, Respiratory Institute, Cleveland Clinic, Cleveland, OH, USA; 16 Division of Pulmonary, Allergy, and Critical Care Medicine, Duke University School of Medicine, Durham, NC; 17 Chest & Lung Center, Ascension Saint Thomas, Nashville, TN; 18 Division of Pulmonary and Critical Care, Northwestern Feinberg School of Medicine, Chicago, IL; 19 Division of Pulmonary, Critical Care, Allergy & Sleep Medicine, Medical University of South Carolina, Charleston, SC; 20 Division of Pulmonary and Critical Care Medicine, Johns Hopkins University School of Medicine, Baltimore, MD; 21 Oxford Respiratory Trials Unit, University of Oxford, Oxford, UK; 22 Oxford NIHR Biomedical Research Centre; 23 Oxford Chinese Academy of Medical Sciences Institute, University of Oxford, Oxford, UK

## Abstract

**Background:**

Millions of pulmonary nodules are identified each year incidentally or via lung cancer screening, many of which require biopsy to distinguish malignancy from benign etiologies. Both navigational bronchoscopy and computed tomography-guided transthoracic biopsy are commonly used for patients undergoing biopsies of peripheral pulmonary nodules, but the relative diagnostic accuracy of these two modalities is unknown.

**Methods:**

In this multicenter, randomized, parallel-group, noninferiority trial, patients with 10–30 mm, intermediate or high risk peripheral pulmonary nodules were randomly assigned to navigational bronchoscopy or transthoracic biopsy at seven centers across the United States. The primary endpoint was diagnostic accuracy, defined as the proportion of biopsies providing a specific diagnosis confirmed through 12 months of clinical follow-up. Secondary outcomes included procedural complications such as the occurrence of pneumothorax.

**Results:**

Among the 234 patients included in the primary analysis, biopsy resulted in a specific diagnosis in 94 of 119 cases (79.0%) in the navigational bronchoscopy group, compared to 81 of 110 cases (73.6%) in the transthoracic biopsy group (absolute difference, 5.4 percentage points; 95% confidence interval, −6.5 to 17.2 (p = 0.003 for noninferiority; p = 0.17 for superiority). Pneumothorax occurred in four patients (3.4%) in the navigational bronchoscopy group and 32 patients (34.8%) in the transthoracic biopsy group with chest tube thoracostomy or admission occurring in one patient (0.9%) and 13 patients (14.1%), respectively.

**Conclusions:**

The diagnostic accuracy of navigational bronchoscopy was noninferior to that of transthoracic biopsy in patients with pulmonary nodules 10–30 mm. (**Trial registration**: ClinicalTrials.gov NCT04250194.)

## INTRODUCTION

Millions of pulmonary nodules are identified annually, many of which require biopsy to distinguish malignancy from benign etiologies.^1–3^ Transthoracic biopsy and navigational bronchoscopy are techniques to biopsy peripheral pulmonary nodules. Transthoracic biopsy is guided by intraprocedural three-dimensional CT (computed tomography) images, providing an accurate diagnosis in up to 90% of cases, but requires passing a needle through the chest wall and pleura causing pneumothorax in up to 25% of cases.^4–8^ During navigational bronchoscopy, nodules are biopsied via catheters guided through peripheral airways based on pre-procedure CT images, without violating the pleurae, causing pneumothorax in 2% of cases^9^. However, navigational bronchoscopy has historically relied on intraprocedural two-dimensional fluoroscopy and diagnostic accuracy has been reported to be as low as 38% with pooled yield of 70% in prior meta-analyses.^9–12^ Recently, intraprocedural three-dimensional imaging (digital tomosynthesis and cone beam CT) have been integrated with navigational bronchoscopy platforms resulting in a similar diagnostic accuracy to transthoracic biopsy in some studies.^13–16^

Diagnostic accuracy estimates for both modalities have been derived from single-arm studies at high risk for selection, referral, and publication biases.^4,11^ No randomized trials have compared navigational bronchoscopy to transthoracic biopsy.

To compare the effect of navigational bronchoscopy to that of transthoracic biopsy on diagnostic accuracy among patients undergoing biopsy of a peripheral pulmonary nodule, we conducted the Navigation Endoscopy to Reach Indeterminate Lung Nodules versus Transthoracic needle Aspiration (VERITAS) trial. We hypothesized the diagnostic accuracy of navigational bronchoscopy would be non-inferior to that of transthoracic biopsy.

## METHODS

### Trial Design and Oversight

VERITAS was an investigator-initiated, multicenter, unblinded, randomized, parallel-group, non-inferiority trial in which navigational bronchoscopy was compared to transthoracic biopsy among patients undergoing biopsy of an indeterminate pulmonary nodule. It was approved by the institutional review board at the Clinical Coordinating Center, Vanderbilt University Medical Center, and all enrolling sites, and was registered at ClinicalTrials.gov before initiation. The protocol and statistical analysis plan (see Supplementary Appendix) were published before the conclusion of enrollment.^17,18^ Central investigators designed the trial and wrote the first manuscript draft; all authors agreed to submit for publication and vouch for the accuracy and completeness of the data and for the fidelity of the trial to the protocol.

### Trial Sites and Patient Population

The trial was conducted at seven sites across the United States. Adults referred for bronchoscopic or transthoracic biopsy of a single, peripheral, indeterminate pulmonary nodule 10 to 30 mm in diameter, with a calculated pre-test probability of malignancy of at least 10%, were eligible.^17,19,20^ Patients were excluded if the nodule could be accessed without navigation (e.g., a central endobronchial lesion), the patient had a separate indication for linear endobronchial ultrasound guided needle aspiration (e.g., mediastinal or hilar adenopathy), empiric treatment with stereotactic radiation was planned regardless of biopsy results, or if biopsy was not feasible by either navigational bronchoscopy or transthoracic biopsy. Details of the trial sites and inclusion and exclusion criteria are provided in the Supplementary Appendix.

### Enrollment, Randomization, and Blinding

Consecutive adults referred for lung nodule biopsy were screened by a local site investigator. After provision of informed consent, chest imaging was centrally reviewed by at least one interventional radiologist and one interventional pulmonologist to confirm the case was technically amenable to both navigational bronchoscopy and transthoracic biopsy. Fully eligible patients were randomly allocated 1:1 to navigational bronchoscopy or transthoracic biopsy, in permuted blocks of variable size, stratified by nodule location (middle third or outer third of the lung), pre-test probability of malignancy (≤50% or >50%^19,20^), and study site, using a cloud-based randomization tool.^21,22^

Clinicians and research personnel were aware of trial-group assignments after randomization given the nature of the intervention. Outcome assessors were blinded to group assignment.

### Trial Intervention

Patients assigned to bronchoscopy underwent electromagnetic navigational bronchoscopy with integrated digital tomosynthesis (ILLUMISITE™ Fluoroscopic Navigation Platform, Medtronic, Minneapolis, MN, USA).^13^ Procedures were planned using a CT scan obtained within 3 months of the procedure. Bronchoscopy was performed under general anesthesia by an interventional pulmonologist with radial endobronchial ultrasound and rapid on-site cytological assessment available. Use of digital tomosynthesis was encouraged when initial radial ultrasound signature was not concentric. The number of biopsies and biopsy tools used were at the discretion of the proceduralist. Fluoroscopy was used to assess for pneumothorax immediately post-procedure.

Patients assigned to transthoracic biopsy underwent CT-guided transthoracic biopsy under local anesthesia with moderate sedation or general anesthesia, per local institutional protocols and clinician and patient preferences. Biopsies were performed by interventional radiologists using dedicated interventional CT scanners. The number of biopsies, size and type of biopsy needle, use of rapid on-site cytologic examination, and method of assessment for post-procedure pneumothorax were at the discretion of the proceduralists.

Patients found to have malignancy on biopsy were referred for oncologic treatment. Cases in which malignancy was not identified underwent guideline recommended follow-up by treating clinicians who managed all care post-biopsy, including any decision to pursue additional invasive diagnostic procedures and cadence of follow-up imaging.

### Data Collection

Demographic and radiographic data were collected by local research personnel. Central investigators recorded technical feasibility for both procedures and nodule location within outer or middle third lung zones.^23^ Procedure data, pathological findings, and complications were collected via medical record review and patient phone call seven days post-procedure.

Biopsies were reviewed by local pathologists for presence of malignancy. Those non-malignant on local review underwent central review by a blinded thoracic pathologist. A committee of three pulmonary nodule experts, blinded to group assignment, centrally reviewed all non-malignant pathology reports and pre-procedural clinical records to adjudicate whether the biopsy was diagnostic or non-diagnostic. A specific benign diagnosis required a unanimous consensus of the three pulmonary nodule experts with cases in which a consensus could not be achieved being adjudicated as non-diagnostic.

Research personnel reviewed medical records for subsequent invasive procedures and a change in the presumed diagnosis of a non-malignant biopsy from enrollment until the first of malignancy diagnosis, nodule regression/resolution, or at least 12 months of radiographic stability by interval CT scan.

### Outcomes

The primary outcome was diagnostic accuracy, defined as the proportion of diagnostic biopsies (showing malignancy or a specific benign diagnosis) with no change in diagnosis through 12 months of clinical follow-up (Supplementary Appendix, Figure S1).^17,24^ Cases canceled because same-day imaging prior to the start of the procedure demonstrated nodule regression were considered diagnostic for that pathway but required confirmation on follow-up diagnostic CT to be considered accurate. Procedures canceled same-day for any other reason except safety (e.g. patient presenting with a new unstable arrhythmia) were considered nondiagnostic, as were any procedures started but not completed.

Secondary outcomes were diagnostic yield (proportion of biopsies considered diagnostic without consideration of clinical follow-up), rate of confident clinical diagnosis, procedural complications, procedure duration, procedural and radiographic features associated with diagnostic yield, need for subsequent nodule biopsy or staging procedure, and radiation exposure (full definitions in Supplementary Appendix).

### Statistical Analysis

Patient demographic and nodule features were summarized for each treatment arm using medians and quartiles for continuous variables and frequencies and percentages for categorical variables. Noninferiority of bronchoscopy was tested using a z-test with noninferiority margin of 10 percentage points (see Supplementary Appendix for noninferiority margin rationale). Diagnostic accuracy of TTNB was assessed at 90%, with noninferiority margin of 10%, one-sided type I error rate of 5%, and power of 80%, yielding a sample of n=112 per group, increased 15% to account for attrition to total n=258. No interim analysis was performed as both biopsy techniques are used in routine clinical care and adjudication of the primary outcome required 12 months.

**Figure 1.**
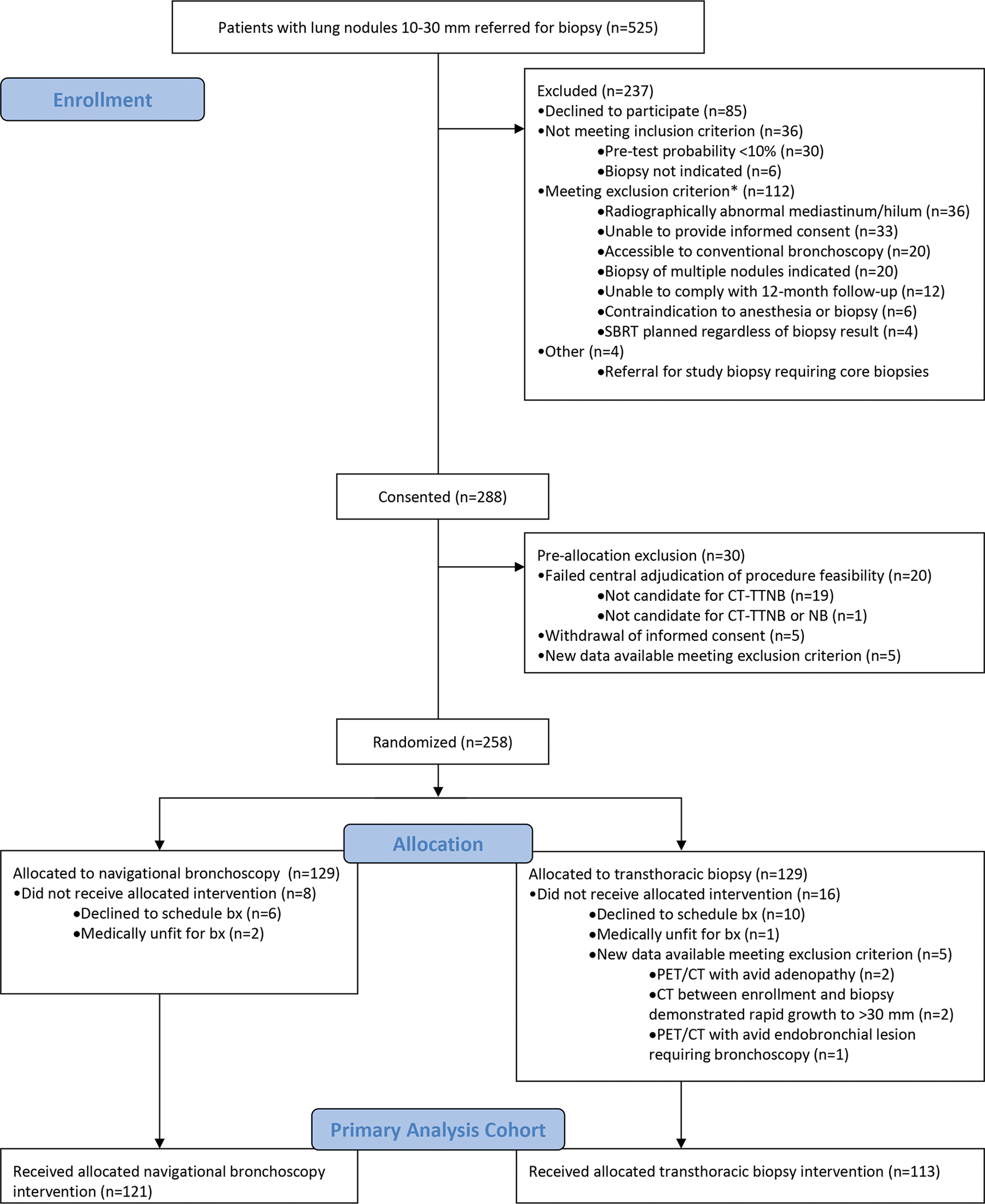
Trial flow. *Some patients met multiple exclusion criteria.

All patients who underwent randomization were included except patients with additional imaging between randomization and biopsy which made them ineligible (e.g. interval imaging showing nodule growth >30 mm or new nodal disease requiring biopsy), patients who did not report for their scheduled trial intervention, patients whose procedure was canceled due to clinical instability prior to the initiation of the trial intervention, and patients missing data on the primary outcome (Figure 1).

We examined whether prespecified baseline variables modified the effect of trial-group assignment on the primary outcome using a logistic regression model with independent variables of the trial group, the proposed effect modifier, and the interaction between the trial group and the proposed effect modifier. Prespecified effect modifiers included nodule location (middle vs. outer third), calculated probability of malignancy (≤50% vs. >50%), nodule size (≤15 mm vs. >15 mm), and presence or absence of a bronchus sign. Sensitivity analyses included (1) an as-randomized analysis including all allocated patients, classifying patients not receiving a study intervention as having not met the primary outcome; (2) a lost to follow-up analysis in which patients lost to follow-up prior to 12 months with specific benign study biopsies were considered either all true negative or all false negative with respect to malignancy, and (3) an analysis limited to patients who underwent nodule biopsy (excluding patients canceled due to nodule regression on same-day imaging).

Between-group differences in secondary outcomes are reported as point estimates and 95% confidence intervals. The widths of the confidence intervals were not adjusted for multiplicity and should not be used to infer definitive differences in treatment effects between the two groups. All the analyses were performed with the use of R software, version 4.4.

## RESULTS

### Patients

Between Sept 16, 2020 and June 14, 2023, 519 patients were assessed for eligibility, 231 patients were excluded or declined, and 288 patients (55.5%) provided consent (Figures 1, S2). The most common reason for exclusion was having a separate clinical indication for bronchoscopy. Among consented patients, 30 were ineligible for randomization after central review (n=20), new imaging meeting an exclusion criterion (n=5), or withdrawal of consent (n=5). A total of 129 patients were randomized to navigational bronchoscopy and 129 patients to transthoracic biopsy. Six patients in the navigational bronchoscopy group and 10 in the transthoracic biopsy group did not report for their scheduled trial intervention, two in the navigational bronchoscopy group and one in the transthoracic biopsy group presented medically unstable and their cases were canceled, and five in the transthoracic biopsy group were excluded based on PET/CT obtained prior to biopsy. A total of 121 of 129 patients (93.8%) assigned to navigational bronchoscopy and 113 of 129 (87.6%) assigned to transthoracic biopsy were included in the primary analysis. Two patients (1.7%) in the navigational bronchoscopy group and three (2.7%) in the transthoracic biopsy group were lost to follow-up before collection of the primary outcome.

Baseline patient demographics and nodule features are shown in Table 1. Median nodule size was 15 mm (IQR, 12–19). Most nodules were solid (82%) and located in the outer third lung zone (88%).

### Primary Outcome

For the primary outcome, diagnostic accuracy, biopsy indicated a specific diagnosis which was confirmed to be accurate through 12 months of clinical follow-up in 94 of 119 cases (79.0%) in the navigational bronchoscopy group compared to 81 of 110 cases (73.6%) in the transthoracic biopsy group (absolute difference, 5.4 percentage points; 95% Confidence Interval [CI], −6.5 to 17.2; p = 0.003 for noninferiority; p = 0.17 for superiority, Table 2). Through 12 months of clinical follow-up, three specific benign cases and one same-day regression case in the transthoracic biopsy group were reclassified to malignant based on subsequent findings, for a false negative rate of 3.6%. No specific benign cases in the navigational bronchoscopy group were re-classified to malignant. The overall prevalence of malignancy through 12 months was 72.1% (74.8% and 69.1% in bronchoscopy and transthoracic biopsy arms, respectively).

**Figure 2.**
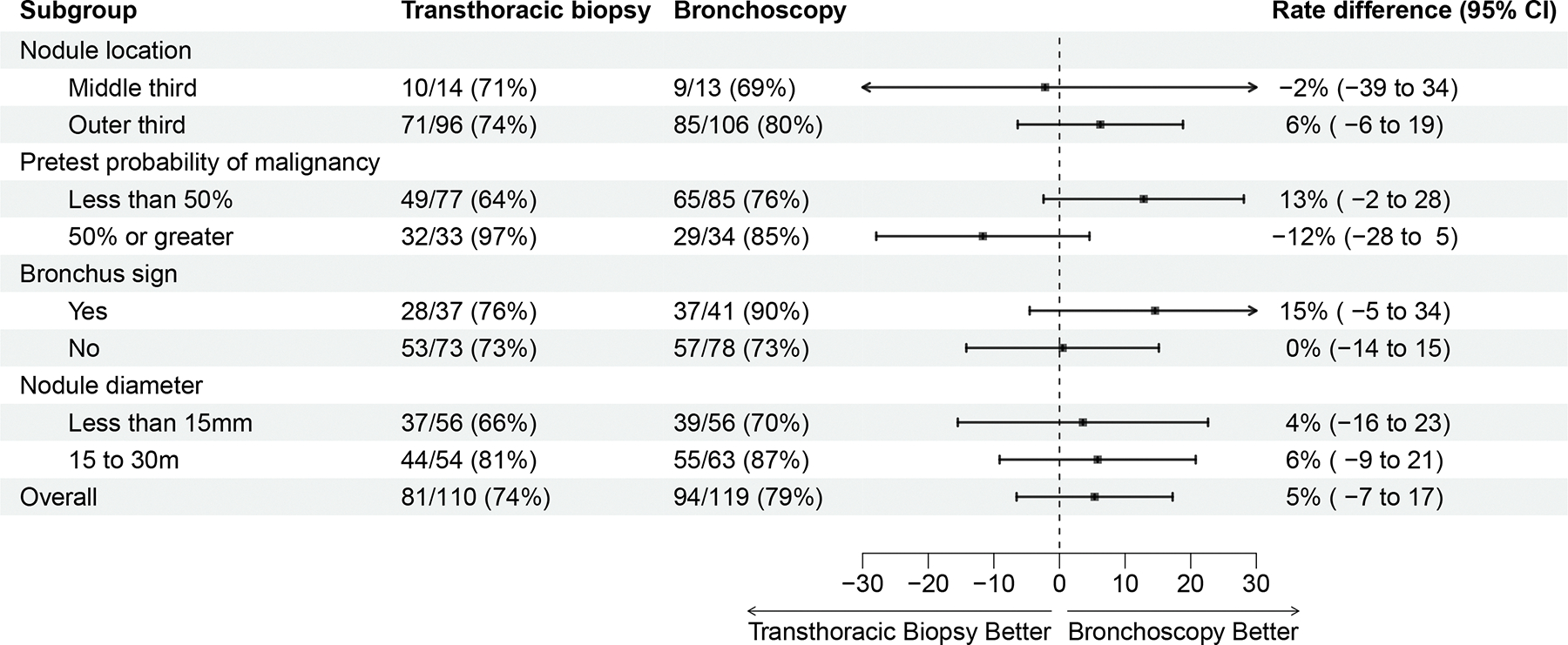
Subgroup analyses of the primary outcome. Shown is the unadjusted mean difference in diagnostic accuracy between patients undergoing navigational bronchoscopy and transthoracic biopsy. The horizontal bars represent the 95% confidence intervals around the mean difference. The number of patients in each group for whom a measure of diagnostic accuracy was available is shown. Pretest probability of malignancy is calculated by validated malignancy risk assessment model.

Findings were similar in sensitivity analyses of the primary outcome (see Supplementary Appendix). No nodule characteristics nor study site appeared to modify the effect of biopsy modality on diagnostic accuracy (Figures 2, S3).

### Secondary Outcomes

Secondary outcomes are detailed in Table 2 and the Supplementary Appendix. Median procedural duration was 36 minutes (IQR, 28 to 47.5) for navigational bronchoscopy and 24.5 minutes (IQR, 13 to 36) for transthoracic biopsy (median difference, 11.5 minutes; 95% CI, 8.2 to 17.6). An invasive diagnostic procedure was pursued subsequent to study biopsy in 13% of cases in both groups (Table 2, Table S9). Procedural and radiographic features associated with diagnostic yield are detailed in the Supplementary Appendix (Table S7, S8).

### Safety Outcomes

A procedural complication occurred in 6 of 121 cases (5.0%) in the navigational bronchoscopy group compared to 33 of 113 cases (29.2%) in the transthoracic biopsy group (absolute risk difference 24.2 percentage points; 95% CI, 15.0 to 35.6, Table 3). Pneumothorax was most common, occurring in 4 patients (3.3%) in the navigational bronchoscopy group and 32 patients (28.3%) in the transthoracic biopsy group (absolute risk difference 25.0 percentage points; 95% CI, 15.3 to 34.8). Pneumothorax requiring chest tube thoracostomy and/or hospital admission occurred in 1 patient (0.8%) and 13 patients (11.5%), respectively (absolute risk difference 10.7 percentage points; 95% CI, 3.7 to 17.6). There were no hemorrhages requiring intervention and no deaths through 12-month follow-up in the primary analysis.

## DISCUSSION

The diagnostic accuracy of navigational bronchoscopy was non-inferior to that of transthoracic biopsy among patients undergoing biopsy of a peripheral pulmonary nodule. Complications were less common during bronchoscopy.

Early diagnosis of lung cancer, which often requires biopsy, offers the best chance for cure. Methodologically rigorous estimates of accuracy and safety of available biopsy modalities are needed to inform the care of patients with indeterminate pulmonary nodules. The results of this trial suggest that navigational bronchoscopy, with similar diagnostic accuracy to transthoracic biopsy but fewer complications, should be the procedure of choice for biopsy of indeterminate lung nodules that appear technically amenable to both approaches.

The performance of navigational bronchoscopy in this trial is comparable to prior studies using the same technique, which have reported yields ranging from 79% to 83%.^13–16^ Transthoracic biopsy performed worse than previously reported. However, the typically higher estimates of diagnostic accuracy for transthoracic biopsy derive from non-comparative and often retrospective studies with high risk of bias and utilize variable outcome definitions.^4,11,24^ Additionally, median nodule size in this trial was relatively small at 15 mm; diagnostic performance of transthoracic biopsy has been notably lower when targeting smaller nodules.^5,25,26^

This trial has several strengths. Patients were screened for eligibility when referred for bronchoscopy or transthoracic biopsy, mitigating the risk of referral bias. Patients were recruited at academic and community centers across different geographic regions and included a representative sample of physicians and patients which strengthens the generalizability of these results (see Table S1). An independent panel adjudicated the technical feasibility of biopsy by both modalities to ensure both were suitable options and to limit selection bias. Pathologists and pulmonary nodule experts adjudicating the primary outcome were blinded to minimize observer bias, and follow-up was robust with minimal losses. The primary outcome was defined conservatively, conforming to current recommendations.^24^ Finally, multiple pre-specified sensitivity analyses of the primary analysis were performed, all of which corroborate the primary analysis demonstrating noninferiority of navigational bronchoscopy to transthoracic biopsy.

This study also has several limitations. While both academic and community centers were included, bronchoscopy was performed by experienced pulmonologists, so these results may not generalize to centers with less expertise. It was not practicable to conceal allocation from proceduralists or patients, though outcome adjudication was blinded. Patients with nodules inaccessible to one or both modalities and those with inner third lung zone nodules were excluded, though this comprised only 20 of 288 (6.9%) provisionally eligible patients, suggesting these trial results generalize to the majority of nodules referred for biopsy. Rapid onsite cytological evaluation was used more commonly during bronchoscopy; whether it affects diagnostic accuracy of navigational bronchoscopy or transthoracic biopsy is uncertain and should be the focus of future study. While pneumothoraces and pneumothoraces requiring hospital admission and/or tube thoracostomy were both more common in the transthoracic biopsy group, these outcomes could have been influenced by universal use of cross-sectional imaging in the transthoracic biopsy group and differences in practice patterns regarding hospitalization based on clinical specialty. Study site did not modify the effect of biopsy modality on diagnostic accuracy, though small samples at some sites limited this analysis. Cost effectiveness was not assessed and should be the topic of future studies. Finally, cases in which same-day pre-procedural imaging revealed substantial regression of the nodule were considered diagnostic in the primary analysis; a sensitivity analysis excluding these cases did not change the results. Diagnostic same-day regression was more frequent in transthoracic biopsy, potentially biasing results in its favor and so strengthens the finding that bronchoscopy is non-inferior to transthoracic biopsy.

In conclusion, the diagnostic accuracy of navigational bronchoscopy is noninferior to that of transthoracic biopsy and leads to fewer complications.

## Supplementary Material

Supplementary Appendix

## Figures and Tables

**Table 1.** Patient and Nodule Characteristics.

Patient Characteristics	NB (n=121)	CT-TTNB (n=113)
Age	66.0 (62.0–72.0)	68.0 (61.0–74.0)
Female sex	57 (47%)	56 (50%)
Race		
White	109 (90%)	104 (92%)
Black	9 (7%)	5 (4%)
Asian	2 (2%)	1 (1%)
Hispanic ethnicity	0	3 (3%)
Comorbidities		
Current or prior malignancy*	46 (38%)	55 (49%)
COPD	49 (40%)	48 (42%)
Coronary artery disease	25 (21%)	16 (14%)
Body mass index	27.6 (23.9–31.5)	27.9 (24.0–31.2)
Tobacco smoking history		
Current	39 (32%)	24 (21%)
Former	55 (45%)	66 (58%)
Pack-years	43.0 (20.0–55.0)	35.0 (20.0–47.0)
**Nodule Characteristics**		
Diameter†	15.0 (12.5–20.0)	14.5 (12.0–18.1)
Lung zone		
Outer third zone	107 (88%)	98 (87%)
Middle third zone	14 (12%)	15 (13%)
Density		
Solid	99 (82%)	94 (83%)
Part-solid	20 (17%)	19 (17%)
Pure GGO	2 (2%)	0 (0%)
Radiographic features		
Spiculated	54 (45%)	64 (57%)
Lobular	36 (30%)	28 (25%)
Smooth	18 (15%)	11 (10%)
Cavitary or cystic component	9 (7%)	4 (4%)
Abutting pleura	14 (12%)	19 (17%)
Bronchus sign present	41 (34%)	38 (34%)
Distance, nodule edge to nearest bronchus	10.0 (0.0–19.0)	12.0 (0.0–20.0)
Distance, nodule edge to pleura	7.0 (0.0–17.2)	7.0 (0.0–20.0)
Pre-test probability of malignancy		
Per enrolling pulmonary nodule clinician	75.0 (50.0–90.0)	75.0 (50.0–90.0)
Per quantitative prediction model ‡	32.0 (20.0–55.8)	30.0 (16.0–51.6)

n (%) or median (IQR). All distance measurements in mm.

* See Table S2 in Supplementary Appendix for details.

† Average of short and long axis in axial plane, mm

‡ Brock^19^ if no PET at time of enrollment; Herder^20^ if PET at time of enrollment

**Table 2.** Primary and secondary outcomes.

	Bronchoscopy (n=121)	Transthoracic Biopsy (n=113)	Difference (95% CI)
**Primary outcome: Diagnostic accuracy**			
Accurate	94/119 (79.0%)	81/110 (73.6%)	5.4 (−6.5 to 17.2)*
Inaccurate	25/119 (21.0%)	29/110 (26.4%)	
False-negative	0	4/110 (3.6%)	
Initially nondiagnostic	25/119 (21.0%)	25/110 (22.7%)	
Lost to follow-up	2 (1.7%)	3 (2.6%)	
**Secondary outcome: Diagnostic yield**			
Diagnostic†	96 (79.3%)	88 (77.6%)	1.5 (−9.9 to 12.8)
Malignant‡	78 (64%)	61 (54%)	
Specific benign	16 (13%)	17 (15%)	
Granulomatous	6 (5%)	10 (9%)	
Organizing pneumonia	1 (1%)	5 (4%)	
Acute neutrophilic inflammation	3 (2.5%)	1 (1%)	
Other specific benign§	6 (5%)	1 (1%)	
Same-day regression¶	2 (1.7%)	10 (8.8%)	
Non-diagnostic	25 (21%)	25 (22%)	
Nonspecific inflammation	10 (8%)	8 (7%)	
Normal lung/airway	9 (7%)	3 (3%)	
Atypia	4 (3%)	2 (2%)	
No biopsies obtained∥	2 (2%)	6 (5%)	
Proceduralist declined**	0	6 (5%)	
**Additional secondary outcomes**			
Underwent procedure††	119 (98%)	97 (86%)	12.5 (4.8 to 20.2)
Duration, minutes	36 (28–47.5)	24.5 (13–36)	11.5 (8.2 to 17.6)
Radiation exposure	9800 mGy*cm^2^ (7190–18850)	659 mGy*cm (253–1276)	‡‡
Intraprocedural ROSE	114/119 (95.8%)	7/97 (7.2%)	89 (81 to 96)
Subsequent invasive diagnostic procedure	16 (13%)	15 (13%)	0.1 (−8.8 to 8.7)
Subsequent invasive staging procedure	0	3 (3%)	3.0 (−40 to 6.7)

n (%) or median (IQR). CI = confidence interval. ROSE = rapid on-site cytological evaluation.

* p = 0.003 for noninferiority; p = 0.17 for superiority

† Biopsies were considered diagnostic if they demonstrated malignant or specific benign pathological findings; see Supplementary Appendix for complete definitions and additional information.

‡ See Table S2 for details of malignant diagnoses.

§ hamartoma (n=4), necroinflammatory (n=1), silicotic nodule (n=1), fibroelastotic scar (n=1)

¶ Substantial regression on same-day cross-sectional imaging intrinsic to the biopsy modality indicating a high probability of benign etiology without biopsy.

∥ Procedure started but no biopsies ultimately obtained (e.g. due to complication).

** Proceduralist declined to proceed despite the case being peer-adjudicated as technically feasible and the patient presented medically fit for biopsy.

†† Minus cases canceled for same-day regression or declined by the proceduralist on the day of procedure.

‡‡ CT scanners and 2D fluoroscopes used in this study reported radiation dose in different, unconvertable units (dose area product and dose length products, respectively), preventing direct comparison across arms.

**Table 3.** Safety outcomes.

	Bronchoscopy (n=121)	Transthoracic Biopsy (n=113)	Difference (95% CI)	P value
Any complication	6 (5.0%)	33 (29.2%)	24.2% (15.0 to 35.6)	<0.01
Pneumothorax	4 (3.3%)	32 (28.3%)	25.0% (15.3 to 34.8)	<0.01
Grade 1–2 (observation or aspiration)	3 (2.5%)	19 (16.8%)	14.3% (6.0 to 22.6)	<0.01
Grade 3–4 (admit +/− chest tube)	1 (0.8%)	13 (11.5%)	10.7% (3.7 to 17.6)	<0.01
Chest tube duration, d	1.0 (1.0 to 1.0)	1.0 (1.0 to 2.0)	0	0.65
Respiratory failure requiring admission	1 (0.8%)	1 (0.9%)	0.1% (−2.5 to 2.4)	0.96
Hemorrhage requiring intervention	0	0	0	
Acute coronary syndrome	1 (0.8%)	0	0.8% (−1.6 to 3.3)	0.33

## References

[1] Gould MK, Tang T, Liu I-LA, Recent Trends in the Identification of Incidental Pulmonary Nodules. Am J Respir Crit Care Med 2015;192(10):1208–14. 26214244 10.1164/rccm.201505-0990OC

[2] National Lung Screening Trial Research Team, Aberle DR, Adams AM, Reduced lung-cancer mortality with low-dose computed tomographic screening. N Engl J Med 2011;365(5):395–409. 21714641 10.1056/NEJMoa1102873 PMC4356534

[3] Koning HJ de, Aalst CM van der, Jong PA de, Reduced Lung-Cancer Mortality with Volume CT Screening in a Randomized Trial. New England Journal of Medicine 2020;382(6):503–13. 31995683 10.1056/NEJMoa1911793

[4] DiBardino DM, Yarmus LB, Semaan RW. Transthoracic needle biopsy of the lung. J Thorac Dis 2015;7(Suppl 4):S304–316. 26807279 10.3978/j.issn.2072-1439.2015.12.16 PMC4700361

[5] Kothary N, Lock L, Sze DY, Hofmann LV. Computed tomography-guided percutaneous needle biopsy of pulmonary nodules: impact of nodule size on diagnostic accuracy. Clin Lung Cancer 2009;10(5):360–3. 19808195 10.3816/CLC.2009.n.049

[6] Ng YL, Patsios D, Roberts H, CT-guided percutaneous fine-needle aspiration biopsy of pulmonary nodules measuring 10 mm or less. Clin Radiol 2008;63(3):272–7. 18275867 10.1016/j.crad.2007.09.003

[7] Vachani A, Zhou M, Ghosh S, Complications After Transthoracic Needle Biopsy of Pulmonary Nodules: A Population-Level Retrospective Cohort Analysis. J Am Coll Radiol 2022;19(10):1121–9. 35738412 10.1016/j.jacr.2022.04.010

[8] Wiener RS, Schwartz LM, Woloshin S, Welch HG. Population-based risk for complications after transthoracic needle lung biopsy of a pulmonary nodule: an analysis of discharge records. Ann Intern Med 2011;155(3):137–44. 21810706 10.1059/0003-4819-155-3-201108020-00003 PMC3150964

[9] Folch EE, Pritchett MA, Nead MA, Electromagnetic Navigation Bronchoscopy for Peripheral Pulmonary Lesions: One-Year Results of the Prospective, Multicenter NAVIGATE Study. J Thorac Oncol 2019;14(3):445–58. 30476574 10.1016/j.jtho.2018.11.013

[10] Ost DE, Ernst A, Lei X, Diagnostic Yield and Complications of Bronchoscopy for Peripheral Lung Lesions. Results of the AQuIRE Registry. Am J Respir Crit Care Med 2016;193(1):68–77. 26367186 10.1164/rccm.201507-1332OC PMC4731617

[11] Nadig TR, Thomas N, Nietert PJ, Guided Bronchoscopy for the Evaluation of Pulmonary Lesions: An Updated Meta-analysis. Chest 2023;163(6):1589–98. 36640994 10.1016/j.chest.2022.12.044 PMC10925546

[12] Wang Memoli JS, Nietert PJ, Silvestri GA. Meta-analysis of Guided Bronchoscopy for the Evaluation of the Pulmonary Nodule. CHEST Journal [Internet] 2012 [cited 2015 Aug 3];142(2). Available from: http://journal.publications.chestnet.org/article.aspx?doi=10.1378/chest.11-1764 10.1378/chest.11-1764 PMC3425336 21980059

[13] Aboudara M, Roller L, Rickman O, Improved diagnostic yield for lung nodules with digital tomosynthesis-corrected navigational bronchoscopy: Initial experience with a novel adjunct. Respirology 2019; 10.1111/resp.13609 31265204

[14] Katsis J, Roller L, Aboudara M, Diagnostic Yield of Digital Tomosynthesis-assisted Navigational Bronchoscopy for Indeterminate Lung Nodules. J Bronchology Interv Pulmonol 2021; 10.1097/LBR.0000000000000766 33734149

[15] Avasarala SK, Roller L, Katsis J, Sight Unseen: Diagnostic Yield and Safety Outcomes of a Novel Multimodality Navigation Bronchoscopy Platform with Real-Time Target Acquisition. Respiration 2021;1–8. 10.1159/000518009 34515222

[16] Low S-W, Lentz RJ, Chen H, Shape-Sensing Robotic-Assisted Bronchoscopy vs Digital Tomosynthesis-Corrected Electromagnetic Navigation Bronchoscopy: A Comparative Cohort Study of Diagnostic Performance. Chest 2022;S0012–3692(22)04032–6. 10.1016/j.chest.2022.10.019 36441041

[17] Lentz RJ, Frederick-Dyer K, Planz VB, Navigational Bronchoscopy vs CT Scan-Guided Transthoracic Needle Biopsy for the Diagnosis of Indeterminate Lung Nodules: Protocol and Rationale for the Navigation Endoscopy to Reach Indeterminate Lung Nodules vs Transthoracic Needle Aspiration, a Randomized Controlled Study Multicenter Randomized Trial. CHEST Pulmonary [Internet] 2024 [cited 2024 Sep 23];2(3). Available from: https://www.chestpulmonary.org/article/S2949-7892(24)00016-3/fulltext 10.1016/j.chpulm.2024.100050 PMC13418960 42548449

[18] Lentz RJ, Frederick-Dyer K, Planz VB, Navigational Bronchoscopy versus Computed Tomography-guided Transthoracic Needle Biopsy for the Diagnosis of Indeterminate Lung Nodules: protocol and rationale for the VERITAS multicenter randomized trial. medRxiv 2023;2023.11.22.23298915.

[19] McWilliams A, Tammemagi MC, Mayo JR, Probability of cancer in pulmonary nodules detected on first screening CT. N Engl J Med 2013;369(10):910–9. 24004118 10.1056/NEJMoa1214726 PMC3951177

[20] Herder GJ, van Tinteren H, Golding RP, Clinical prediction model to characterize pulmonary nodules: validation and added value of 18F-fluorodeoxyglucose positron emission tomography. Chest 2005;128(4):2490–6. 16236914 10.1378/chest.128.4.2490

[21] Harris PA, Taylor R, Minor BL, The REDCap consortium: Building an international community of software platform partners. Journal of Biomedical Informatics 2019;95:103208. 31078660 10.1016/j.jbi.2019.103208 PMC7254481

[22] Harris PA, Taylor R, Thielke R, Payne J, Gonzalez N, Conde JG. Research electronic data capture (REDCap)—A metadata-driven methodology and workflow process for providing translational research informatics support. Journal of Biomedical Informatics 2009;42(2):377–81. 18929686 10.1016/j.jbi.2008.08.010 PMC2700030

[23] Thiboutot J, Lee HJ, Silvestri GA, Study Design and Rationale: A Multicenter, Prospective Trial of Electromagnetic Bronchoscopic and Electromagnetic Transthoracic Navigational Approaches for the Biopsy of Peripheral Pulmonary Nodules (ALL IN ONE Trial). Contemp Clin Trials 2018;71:88–95. 29885373 10.1016/j.cct.2018.06.007

[24] Gonzalez AV, Silvestri GA, Korevaar DA, Assessment of Advanced Diagnostic Bronchoscopy Outcomes for Peripheral Lung Lesions: A Delphi Consensus Definition of Diagnostic Yield and Recommendations for Patient-centered Study Designs. An Official American Thoracic Society/American College of Chest Physicians Research Statement. Am J Respir Crit Care Med 2024;209(6):634–46. 38394646 10.1164/rccm.202401-0192ST PMC10945060

[25] Liu H, Yao X, Xu B, Zhang W, Lei Y, Chen X. Efficacy and Safety Analysis of Multislice Spiral CT-Guided Transthoracic Lung Biopsy in the Diagnosis of Pulmonary Nodules of Different Sizes. Comput Math Methods Med 2022;2022:8192832. 36060660 10.1155/2022/8192832 PMC9436531

[26] Huang M-D, Weng H-H, Hsu S-L, Accuracy and complications of CT-guided pulmonary core biopsy in small nodules: a single-center experience. Cancer Imaging 2019;19(1):51. 31337425 10.1186/s40644-019-0240-6 PMC6651998

