# Supplementary material for: Navigational Bronchoscopy versus Transthoracic Biopsy for Lung Nodules
Source: N Engl J Med. Author manuscript; Available in PMC 2025 Nov 23. (PMC12640718; doi:10.1056/NEJMoa2414059)
Supplement: Supplementary Appendix [file NIHMS2119644-supplement-Supplementary_Appendix.pdf]

Supplementary Appendix: VERITAS

VERITAS Sites and Investigators 3
Supplementary Methods 4
Full Inclusion and Exclusion Criteria 4
Outcome definitions 5
Figure S1. Diagnostic outcomes schematic. 6
Bayesian (protocol) versus Frequentist (primary manuscript) analysis 7
Noninferiority margin determination 7
Supplementary Results 8
Figure S2. Sankey Trial Flow Diagram 8
Supplemental demographic information 9
Table S1. Representativeness of study participants. 9
Table S2. Trial participant malignancy-related history 10
Primary outcome: diagnostic accuracy 11
Table S3. Sub-group analyses of the primary outcome (diagnostic accuracy) 11
Sensitivity Analyses of the primary outcome (diagnostic accuracy) 12
(1) As-randomized analysis 12
(2) Lost to follow-up analyses 12
(3) Analysis limited to those who underwent nodule biopsy 12
Table S4. Results of sensitivity analyses of the primary outcome (diagnostic accuracy) 12
Table S5. Patient and nodule characteristics, as-randomized cohort. 13
Figure S3. Heterogeneity of treatment effect by study site 14
Secondary outcomes 15
Table S6. Malignant diagnoses established by study biopsy. 15
Diagnostic Yield (secondary outcome) 16
Figure S4. Subgroup analyses of diagnostic yield (secondary outcome). 16
Sensitivity analysis of diagnostic yield (secondary outcome) in as-randomized cohort 16
Procedural and radiographic features associated with diagnostic yield (secondary outcome) 17
Table S7. Bronchoscopy procedure details and association with diagnostic yield. 17
Table S8. Transthoracic biopsy procedure details and association with diagnostic yield. 18
Additional invasive diagnostic and staging procedures (secondary outcome) 19
Table S9. Additional invasive diagnostic and staging procedures. 19
Confident clinical diagnosis (secondary outcome) 19

Author Contributions 20
Supplementary References 21

VERITAS Sites and Investigators

Site	Site PI	Co-Investigators
Vanderbilt University Medical Center<br>Nashville, TN, USA<br>(central coordinating center)	Fabien Maldonado	Robert Lentz, Katherine Frederick-Dyer, Virginia B. Planz, Otis Rickman, Sameer Avasarala, See-Wei Low, Cristina Salmon, Jennifer Duke, Samira Shojaee, Charla Walston, Joyce Johnson
Saint Luke's Hospital of Kansas City<br>Kansas City, MO, USA	Matthew Aboudara	Timothy Saettele, Jennifer Buckley, Brandt Wible, Nathan Saucier
Duke University Medical Center<br>Durham, NC, USA	Kamran Mahmood	Momen Wahidi
University of California, San Diego<br>San Diego, CA, USA	George Cheng	Kelly Ball, Jeet Minocha, Jonas Redmond
Rush University Medical Center<br>Chicago, IL, USA	James Katsis	Jordan Tasse, Sarah Lynne
Medical College of Wisconsin<br>Milwaukee, WI, USA	Jonathan Kurman	Elizabeth Malsin, Steven Verga, Antonio Sosa Lozano
Kootenai Health<br>Coeur d'Alene, ID, USA	Todd Hoopman	

Supplementary Methods

Full Inclusion and Exclusion Criteria

Inclusion criteria	1. ≥ 18 years of age at time of signing informed consent.<br>2. Referred for biopsy of a single IPN with the following characteristics:<br>a) Pre-test probability of malignancy of at least 10% using a validated clinical prediction model, which is either:<br>a. The Brock model[1] if no PET data are available, or<br>b. The Herder model[2] if PET data are available<br>b) Size between 10 and 30 mm (long diameter, inclusive).<br>c) *Peripheral in location, defined as occupying the middle or outer third lung zones, determined by segmentation using “CT Pulmo 3D” workflow (OsiriX, Pixmeo, Bernex, Switzerland).[3]<br>d) *Technically amenable to both NB and CT-guided biopsy as confirmed by independent central adjudication panels with expertise in IPN biopsy, one comprised of interventional pulmonologists and one interventional radiologists, with the following pre-specified reasons for which a case can be deemed not technically amenable:<br>a. NB: no airway within 3 cm of the IPN<br>b. CT-TTNB: approach would require crossing a fissure or a bulla<br>Procedures may be deemed unsuitable for other reasons by either group with specific rationale recorded in the research record.
Exclusion criteria	1. Central IPN that is accessible via endobronchial biopsy or linear endobronchial ultrasound-guided transbronchial needle aspiration.<br>2. Clinical indication for simultaneous biopsy of multiple IPNs.<br>3. Radiographically abnormal mediastinal or hilar lymph nodes for which endobronchial ultrasound-guided sampling is clinically indicated.<br>4. Stereotactic body radiation therapy planned even if biopsies show no evidence of malignancy.<br>5. Contraindication to biopsy or deep sedation/general anesthesia.<br>6. Inability or unwillingness to comply with study follow-up schedule.<br>7. Previous randomization in this study.<br>8. Inability to provide documented informed consent.<br>9. Pregnant or nursing.

*Location in the middle or outer third and technical amenability to sampling via both modalities will be confirmed by central radiologic and interventional pulmonary and interventional radiology adjudication panels prior to randomization.

Outcome definitions

Primary endpoint

The primary endpoint was diagnostic accuracy, defined as the proportion of cases yielding a specifically diagnostic result which remains accurate through 12 months of clinical follow-up, divided by the total number of cases in the analyzed cohort allocated to that modality, comparing the navigation bronchoscopy to the CT-guided biopsy pathway.

Biopsies meeting the following criteria were considered accurate:

- Malignant
- Diagnostic specific benign pathology (per diagnostic yield definition below), if:
  - The nodule markedly regresses or resolves on follow-up imaging, OR
  - Subsequent resection demonstrates the same pathology, OR
  - A persistent nodule has not been diagnosed as malignant, AND
  - There are no plans for repeat invasive diagnostic procedures through 12 months follow-up.

Secondary endpoints

1. Diagnostic yield: the proportion of cases in which the biopsy procures a pathological result which readily explains the presence of a nodule and allows for immediate confident management of the patient. The following were pre-specified as diagnostic:
   a. Malignant
   b. Specific benign findings which readily explain the presence of a nodule
      i. Organizing pneumonia
      ii. Granulomatous inflammation
      iii. Frank purulence or robust neutrophilic inflammation
      iv. Other specific findings, with agreement by lung nodule physician and thoracic pathologist (e.g. findings suggesting amyloidoma or hamartoma).

   Biopsies with normal lung parenchyma or airway components, atypia not diagnostic of malignancy, and mild or nonspecific inflammatory changes were always considered nondiagnostic.
2. Confident clinical diagnosis: the proportion of cases which yield a confident clinical diagnosis, which also includes any added yield from endobronchial ultrasound-guided mediastinal and/or

hilar lymph node biopsies or microbiologic studies which yield an explanation for a nodule despite non-diagnostic biopsy pathological findings.

3. Rate of biopsy complications, using standard clinical definitions and CTCAE grading.
4. Procedure duration, defined as the interval between first introduction of the navigation catheter to initiate airway registration to bronchoscope removal in the navigational bronchoscopy arm and the interval from first transthoracic needle introduced to last transthoracic needle removed for transthoracic biopsy.
5. Procedural or radiographic features associated with diagnostic yield.
6. Need for additional nodule biopsy.
7. Need for additional staging procedure.
8. Radiation exposure.

Figure S1. Diagnostic outcomes schematic.

PNA: pneumonia. Path: pathology. CT: computed tomography. f/u: follow-up. Dx: diagnosis.

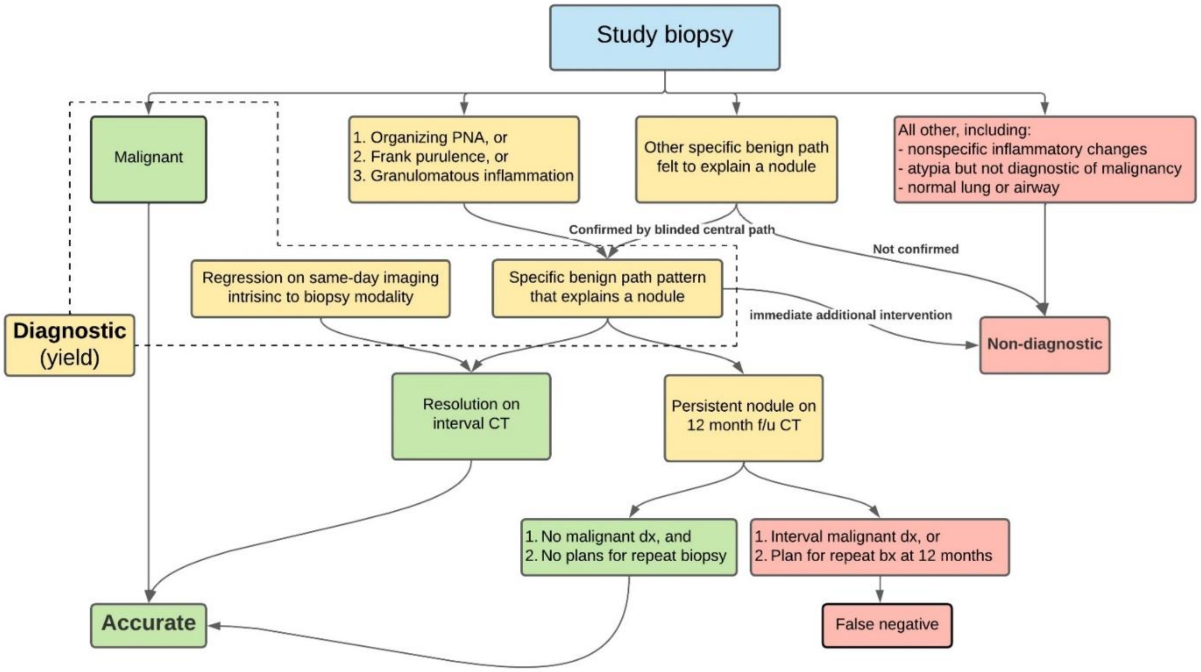

Bayesian (protocol) versus Frequentist (primary manuscript) analysis

The original trial protocol specified a Bayesian analysis of the primary outcome (diagnostic accuracy) and main secondary outcome (diagnostic yield). NEJM statistical and style guidelines require uniformly Bayesian or uniformly frequentist analyses. Per this requirement, we have transitioned to a uniformly Frequentist analysis for the primary manuscript. We do note that a Frequentist power calculation was used to begin the Bayesian sample size determination and ultimately the sample size suggested by the original power calculation was used in this trial, so it is appropriately powered in the Frequentist paradigm. We stress that our primary outcome met noninferiority within both Frequentist and Bayesian analyses in primary and all pre-specified sensitivity analyses of the primary outcome (diagnostic accuracy).

Noninferiority margin determination

A noninferiority margin of 10% was chosen. Contemporary NB utilizing digital tomosynthesis for CT-body divergence correction has been associated with diagnostic accuracy estimates around 80%, while the pooled diagnostic accuracy estimate of transthoracic biopsy in large meta-analyses is around 90%.[4–7] A diminution in diagnostic accuracy by less than 10% with NB additionally seems balanced by the greater than 20% increase in pneumothorax risk and ~10% hospital admission rate in transthoracic biopsy versus NB.[7–9] Please see the published protocol paper for additional details.[10]

Supplementary Results

Figure S2. Sankey Trial Flow Diagram

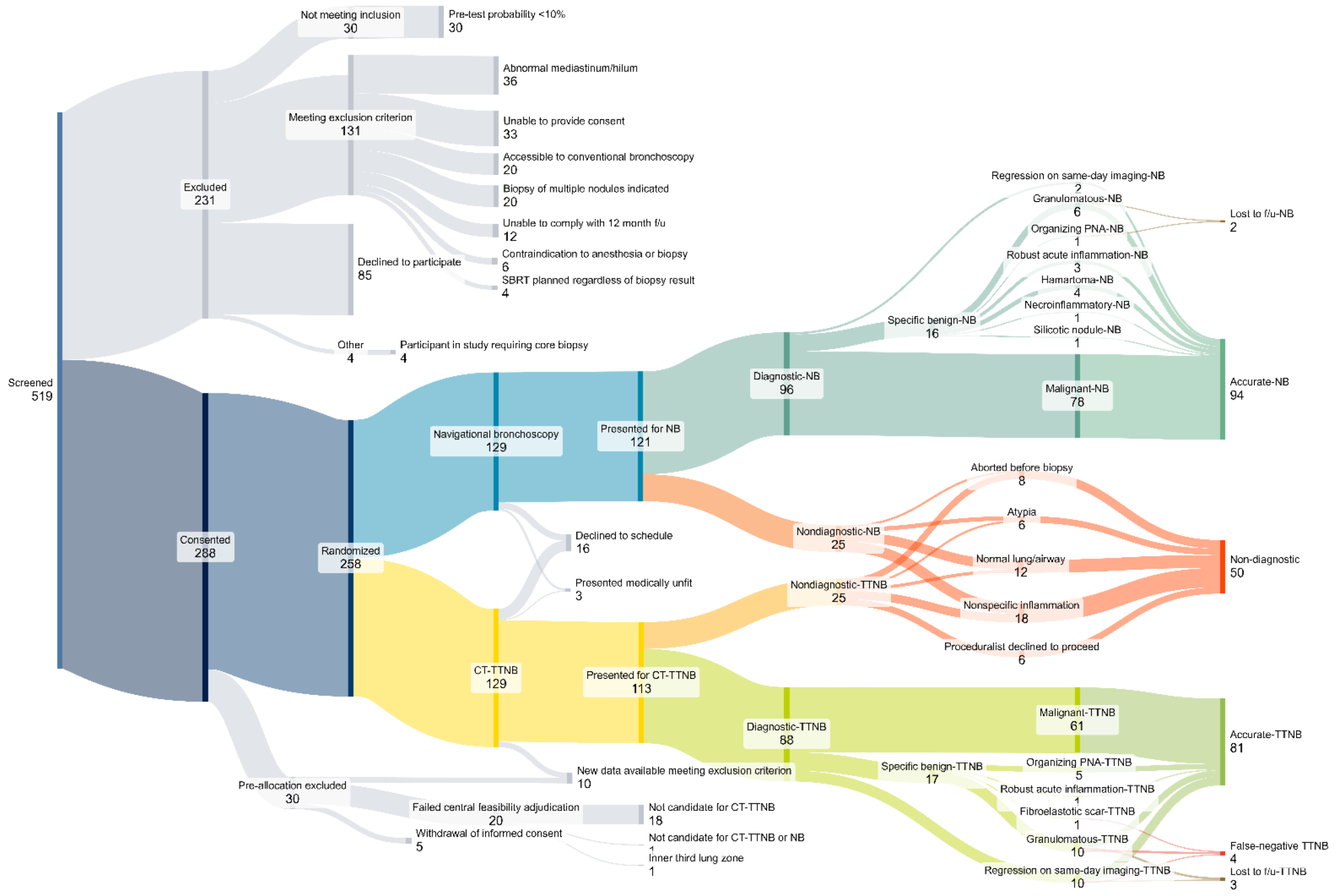

Supplemental demographic information

Table S1. Representativeness of study participants.

Condition under investigation	Indeterminate pulmonary nodules
Special considerations related to	
Sex and gender	The incidence of lung nodule detection appears similar in males and females.
Age	In large US health-system derived data, the mean age of patients with new diagnosis of a lung nodule was 63 +/- 14 years.[11] In an analogous large European cohort, median age was 60 years.[12]
Race or ethnic group	There are no clear data indicating whether lung nodule incidence varies across racial and ethnic groups adjusted for population sizes. In large trials involving lung nodules or lung cancer screening, such as NLST, 91% of participants were white, 4.5% black, 2% Asian, and remainder Native American, Pacific Islander, or from multiracial background; 2% reported Hispanic ethnicity.[13]
Smoking history	In large health-system derived data, among those with lung nodule(s) identified by CT scan, 44% were never smokers, 36% former smokers, and 10% current smokers.[11] In an analogous large European cohort, 39% were never smokers, 47% were former smokers, and 14% were current smokers.[12]
Geography	Regional incidence of new lung nodule detection is poorly characterized in the literature, though it is likely to exist, given variation in the prevalence of underlying conditions which cause lung nodules (namely, lung cancer and endemic fungal or mycobacterial disease).
Overall representativeness of this trial	Median age of participants in this trial of mid 60s years old, similar proportion of male and female participants, and rates of current or prior smoking are similar to demographic characteristics described in large population-level nodule datasets. While degree of variation of lung nodule incidence across racial and ethnic groups is poorly characterized, the racial and ethnic breakdown in this trial is similar to large trials related to lung nodules. There is almost certainly geographic variation in lung nodule incidence related to underlying prevalence of lung cancer and endemic infections; this trial included sites in multiple regions across the United States to recruit a geographically-representative sample. Considering all these elements, the sample in this trial appears similar to the population of interest.

Table S2. Trial participant malignancy-related history

Malignancy	Bronchoscopy (n=121)	TTNB (n=113)
Current/active malignancy	14	20
NSCLC, adenocarcinoma	1	0
NSCLC, squamous cell	0	1
NSCLC, NOS	0	1
Mesothelioma	0	1
Bladder/urothelial	2	5
Breast	2	1
Cervical	0	2
CLL	1	1
Colorectal	1	3
H&N squamous cell	2	2
H&N adenoid cystic	1	0
Hepatic	1	0
Melanoma	0	1
Pancreatic	0	2
Prostate	0	1
Renal	1	0
Sarcoma	2	1
Prior malignancy	32	35
NSCLC, adenocarcinoma	3	5
NSCLC, squamous cell	0	3
Carcinoid, lung primary	0	1
Bladder/urothelial	0	3
Breast	3	3
CNS primary	0	1
Colorectal	1	2
H&N, squamous cell	6	4
Lymphoma	2	3
Melanoma	5	2
Ovarian	1	0
Pancreatic	2	2
Prostate	4	5
Renal	3	3
Sarcoma	2	1
Testicular	0	1
Thyroid	1	1
Vulvar carcinoma	1	1

NSCLC = nonsmall cell lung cancer; H&N = head and neck; CLL = chronic lymphocytic leukemia. Some had both active and prior malignancies, and some had more than one prior malignancy.

Primary outcome: diagnostic accuracy

Table S3. Sub-group analyses of the primary outcome (diagnostic accuracy)

Subgroup	Bronchoscopy	Transthoracic biopsy	Difference (95% CI)
Nodule location			
Middle third	9/13 (69%)	10/14 (71%)	-2% (-39 to 24)
Outer third	85/106 (80%)	71/96 (74%)	6% (-6 to 19)
Pre-test probability of malignancy			
Less than 50%	65/85 (76%)	49/77 (64%)	13% (-2 to 28)
50% or greater	29/34 (85%)	32/33 (97%)	-12% (-28 to 5)
Bronchus sign			
Yes	37/41 (90%)	28/37 (76%)	15% (-5 to 34)
No	57/78 (73%)	53/73 (73%)	0% (-14 to 15)
Nodule diameter			
<15 mm	39/56 (70%)	37/56 (66%)	4% (-16 to 23)
15-30 mm	55/63 (87%)	44/54 (81%)	6% (-9 to 21)
Overall	94/119 (79%)	81/110 (74%)	5% (-7 to 17)

Mean differences with 95% confidence intervals, bronchoscopy minus transthoracic biopsy. Pre-test probability of malignancy is per validated malignancy risk assessment model. See also Figure 2 in the main manuscript.

Sensitivity Analyses of the primary outcome (diagnostic accuracy)

The following headings describe pre-specified analytic cohorts in which sensitivity analyses of the primary outcome were performed. The results of these analyses are summarized in Table S4 which follows.

(1) As-randomized analysis

This sensitivity analysis of the primary outcome included all allocated patients (n=129 in both arms), with diagnostic failure assigned to patients who did not receive a trial intervention. See Table S5 below for demographic characteristics for this cohort.

(2) Lost to follow-up analyses

These sensitivity analyses of primary outcome diagnostic accuracy included the five patients with specific benign findings at study biopsy who were lost to follow-up before 12 months and therefore were missing primary outcome data. These missing primary outcomes were designated A) all inaccurate (false negative for malignancy) and B) all accurate (true negative for malignancy), respectively, in the following analyses.

(3) Analysis limited to those who underwent nodule biopsy

In this sensitivity analysis of primary outcome diagnostic accuracy, only patients who underwent biopsy were included. Patients with biopsies deferred due to nodules regressing on same-day imaging intrinsic to the biopsy procedure and those who presented for biopsy but the proceduralist declined to proceed for reasons other than a medically unfit patient were excluded from this analysis.

Table S4. Results of sensitivity analyses of the primary outcome (diagnostic accuracy)

Sensitivity cohort	Bronchoscopy	TTNB	Difference (95% CI)
1) As-randomized	94/129 (72.9%)	81/129 (62.8%)	10.1 (-2 to 22.2)
2a) Lost to f/u = inaccurate	94/121 (77.7%)	81/113 (71.7%)	6.0 (-6 to 18)
2b) Lost to f/u = accurate	96/121 (79.3 %)	84/113 (74.3%)	5.0 (-7 to 17)
3) Underwent biopsy	92/119 (77.3 %)	73/97 (75.3%)	2.1 (-10.3 to 14.4)

Table S5. Patient and nodule characteristics, as-randomized cohort.

**Patient Characteristics**	**NB** (n=129)	**CT-TTNB** (n=129)
Age	66.0 (62.0-72.0)	68.0 (61.0-74.0)
Female sex	62 (48%)	66 (51%)
Race		
White	115 (89%)	117 (91%)
Black	11 (9%)	5 (4%)
Asian	2 (2%)	1 (1%)
Hispanic ethnicity	0	4 (3%)
Comorbidities		
Current or prior malignancy	50 (39%)	60 (47%)
COPD	52 (40%)	55 (43%)
Coronary artery disease	26 (20%)	18 (14%)
Body mass index	27.4 (23.9-31.5)	27.8 (24.0-31.2)
Tobacco smoking history		
Current	43 (33%)	31 (24%)
Former	58 (45%)	72 (56%)
Pack-years	43.0 (20-55)	35.0 (20-50)
**Nodule Characteristics**		
Diameter, mm	15.0 (12.5-20)	14.0 (11.5-18)
Lung zone		
Outer third zone	114 (88%)	114 (88%)
Middle third zone	15 (12%)	15 (12%)
Density		
Solid	104 (81%)	109 (84%)
Part-solid	22 (17%)	20 (16%)
Pure GGO	3 (2%)	0 (0%)
Radiographic features		
Spiculated	57 (44%)	71 (55%)
Lobular	36 (28%)	33 (26%)
Smooth	19 (15%)	12 (9%)
Cavitary or cystic component	11 (9%)	9 (7%)
Abutting pleura	15 (12%)	21 (16%)
Bronchus sign present	42 (33%)	41 (32%)
Distance, nodule edge to nearest bronchus, mm	10.0 (0-19)	12.0 (0-20)
Distance, nodule edge to pleura, mm	7.0 (0-18)	6.0 (0-18)
Pre-test probability of malignancy		
Per enrolling lung nodule clinician	75.0 (50-90)	75.0 (50-90)
Per quantitative prediction model	33.1 (20-56)	29.0 (16-52)

Figure S3. Heterogeneity of treatment effect by study site

Site	Transthoracic Biopsy	Bronchoscopy	Diagnostic Accuracy Difference
Site 1	51/71	62/77	
Site 2	16/19	18/22	
Site 3	7/7	5/7	
Site 4	3/6	5/5	
Site 5	2/3	2/3	
Site 6	0/2	1/3	
Site 7	2/2	1/2	
Overall	81/110	94/119	

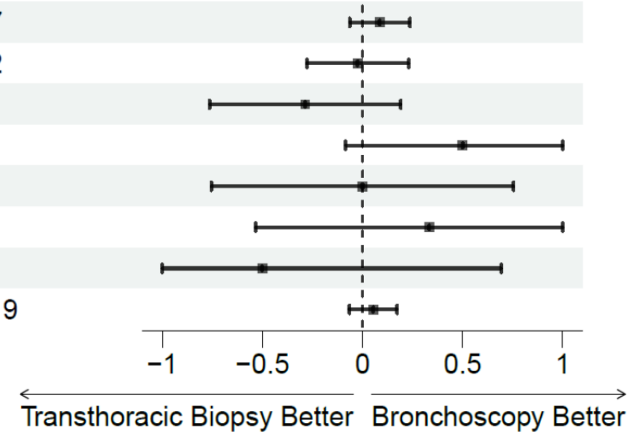

Secondary outcomes

Table S6. Malignant diagnoses established by study biopsy.

	**Bronchoscopy** (n=121)	**Transthoracic Biopsy** (n=113)
Diagnostic	96 (79.3%)	88 (77.6%)
Malignant	78 (64%)	61 (54%)
Lung primary	62 (51%)	40 (35%)
NSCLC (adenocarcinoma)	33 (27%)	24 (21%)
NSCLC (squamous cell carcinoma)	14 (12%)	9 (8%)
NSCLC, not otherwise specified	6 (5%)	1 (0.9%)
Small cell lung carcinoma	4 (3.3%)	1 (0.9%)
Carcinoid	3 (2.5%)	1 (0.9%)
Large cell neuroendocrine carcinoma	2 (1.7%)	1 (0.9%)
NSCLC, mixed adenosquamous	0	3 (2.7%)
Metastatic	16 (13%)	21 (19%)
Acinic cell carcinoma	0	1 (0.9%)
Adenoid cystic carcinoma	1 (0.8%)	0
Colorectal adenocarcinoma	0	3 (2.7%)
Follicular thyroid carcinoma	1 (0.8%)	0
Hepatocellular carcinoma	0	1 (0.9%)
Lymphoma (marginal zone)	0	1 (0.9%)
Mammary carcinoma	2 (1.7%)	1 (0.9%)
Melanoma	2 (1.7%)	3 (2.7%)
Pancreatic adenocarcinoma	0	2 (1.8%)
Prostate adenocarcinoma	1 (0.8%)	0
Renal cell carcinoma	1 (0.8%)	2 (1.8%)
Sarcoma	5 (4%)	3 (2.7%)
Squamous cell carcinoma (head/neck)	1 (0.8%)	1 (0.9%)
Squamous cell carcinoma (chest wall primary)	0	1 (0.9%)
Urothelial carcinoma	2 (1.7%)	2 (1.8%)

This table supplements Table 2 in the main manuscript with additional details of malignant diagnoses established by study biopsies.

Diagnostic Yield (secondary outcome)

Diagnostic yield was met in 96 of 121 cases (79.3%) in the bronchoscopy group compared to 88 of 113 cases (77.6%) in the transthoracic biopsy group (absolute difference, 1.5 percentage points; 95% CI, -9.9 to 12.8). Navigational bronchoscopy revealed malignancy in 78 (64.4%) of cases, a specific benign finding in 16 (13.2%), diagnostic regression on same-day imaging in 2 (1.7%), and non-diagnostic findings in 25 (20.7%). Transthoracic biopsy demonstrated malignancy in 61 (54.0%) of cases, a specific benign finding in 17 (15.0%), diagnostic regression on same-day imaging in 10 (8.8%), and non-diagnostic findings in 25 (22.1%). See also Table 2 in the main manuscript for details on biopsy pathological findings.

Figure S4. Subgroup analyses of diagnostic yield (secondary outcome).

Subgroup	Transthoracic biopsy	Bronchoscopy	Rate difference (95% CI)
Nodule location			
Middle third	11/15 (73%)	10/14 (71%)	−2% (−36 to 33)
Outer third	77/98 (79%)	86/107 (80%)	2% (−10 to 14)
Pretest probability of malignancy			
Less than 50%	55/79 (70%)	66/86 (77%)	7% (−8 to 22)
50% or greater	33/34 (97%)	30/35 (86%)	−11% (−27 to 4)
Bronchus sign			
Yes	30/38 (79%)	37/41 (90%)	11% (−7 to 30)
No	58/75 (77%)	59/80 (74%)	−4% (−18 to 11)
Nodule diameter			
Less than 15mm	39/56 (70%)	40/57 (70%)	1% (−17 to 18)
15 to 30m	48/56 (86%)	56/64 (88%)	2% (−12 to 16)
Overall	88/113 (78%)	96/121 (79%)	1% (−10 to 13)

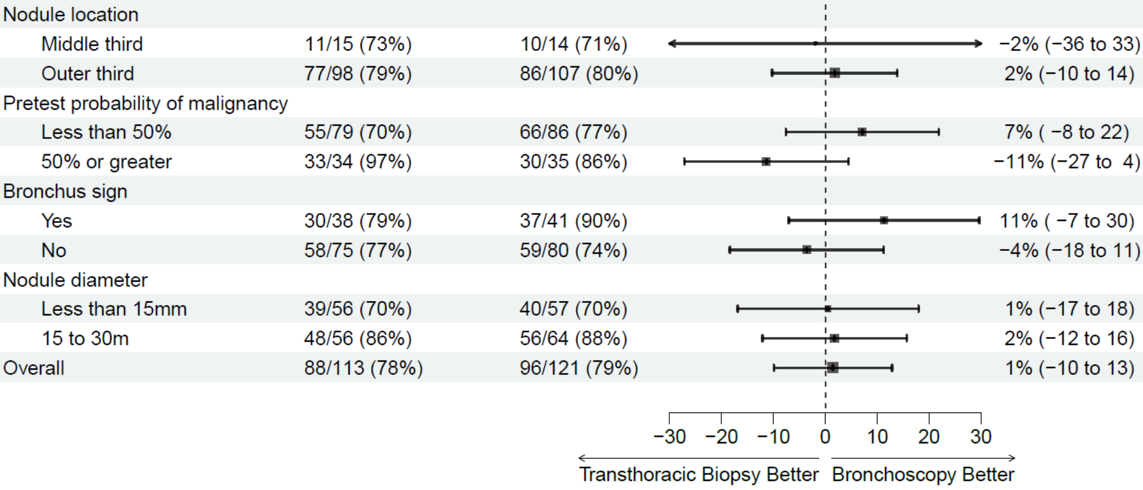

Shown is the unadjusted mean difference in diagnostic yield between patients undergoing navigational bronchoscopy and transthoracic biopsy. The horizontal bars represent the 95% confidence intervals around the mean difference. Pretest probability of malignancy here is per validated malignancy risk assessment model.

Sensitivity analysis of diagnostic yield (secondary outcome) in as-randomized cohort

Diagnostic yield was also analyzed in the as-randomized cohort, including all patients who received a study allocation (n=129 per arm). Navigational bronchoscopy was diagnostic in 96 of 129 cases (74.4%) compared to 88 of 113 cases (68.2%) in the transthoracic biopsy group (absolute difference, 6.2 percentage points; 95% CI, -5.6 to 18; p=0.002 for noninferiority; p=0.135 for superiority).

Procedural and radiographic features associated with diagnostic yield (secondary outcome)

Radiographic features traditionally associated with higher diagnostic yield, including larger size, bronchus sign, and more peripheral location, were assessed. Diagnostic yield for bronchoscopy and transthoracic biopsy for nodules 15-30 mm and <15 mm was 87.5% and 85.7% and 70% and 70%, respectively. Diagnostic yield of bronchoscopy with bronchus sign was 90% vs. 74% without bronchus sign. Diagnostic yield for transthoracic biopsy with bronchus sign was 78% vs. 77% without bronchus sign. Diagnostic yield of bronchoscopy and transthoracic biopsy was 80% and 78% in the outer third of the lung; diagnostic yield was 71% and 73%, respectively, for these procedures in the middle third of the lung. See also Figure S4.

Procedure-related factors are detailed in Tables S7 (bronchoscopy) and S8 (TTNB) below.

Table S7. Bronchoscopy procedure details and association with diagnostic yield.

	Overall n=119	Diagnostic n=94	Nondiagnostic n=25
Visible on 2D fluoro	79 (66%)	68 (72%)	11 (44%)
Initial REBUS signature			
Concentric	20 (17%)	20 (21%)	0
Eccentric	39 (33%)	30 (32%)	9 (36%)
Could not localize	47 (39%)	32 (34%)	15 (60%)
Digital tomosynthesis utilized	96 (81%)	71 (76%)	25 (100%)
Nodule visible on digital tomosynthesis	78 (66%)	60 (64%)	18 (72%)
TBNA performed	116 (97%)	93 (99%)	23 (92%)
TBNA needle passes	8.0 (6.0-8.2)	8.0 (6.0-8.0)	8.0 (6.5-10.0)
Forceps TBB performed	76 (64%)	64 (68%)	12 (48%)
Forceps TBB pass count	5.0 (4.0-6.0)	5.0 (4.0-6.0)	5.0 (4.0-8.0)
Peripheral brushing with standard brush performed	22 (18%)	17 (18%)	5 (20%)
Triple needle brushing performed	2 (2%)	2 (2%)	0
GenCut biopsy tool used	3 (3%)	1 (1%)	2 (8%)
Peripheral wash via navigation catheter performed	31 (26%)	22 (23%)	9 (36%)
Any specimen cultured	25 (21%)	15 (16%)	10 (40%)
ROSE assessment			
Malignant	55 (46%)	55 (59%)	0
Inadequate for immediate malignant diagnosis	58 (49%)	36 (38%)	22 (88%)
Not utilized	5 (4%)	2 (2%)	3 (12%)
Navigational bronchoscopy duration, minutes	36.0 (28.0, 47.5)	34.0 (27.0, 44.0)	44.5 (35.0, 63.5)
Bronchoscopist assessed successful biopsy	114 (96%)	92 (98%)	22 (88%)
Linear endobronchial ultrasound examination performed	101 (85%)	79 (84%)	22 (88%)
At least one station sampled	70 (59%)	56 (60%)	14 (56%)
Nodal malignancy identified	2 (1.7%)	2 (2.1%)	0

n (%) or median (IQR)

Table S8. Transthoracic biopsy procedure details and association with diagnostic yield.

	Overall n=97	Diagnostic n=78	Nondiagnostic n=19
Coaxial needle gauge			
<19	6 (6%)	4 (5%)	2 (11%)
19	75 (77%)	64 (82%)	11 (58%)
20	12 (12%)	9 (12%)	3 (16%)
Not used	4 (4%)	1 (1%)	3 (16%)
FNA performed	6 (6%)	4 (5%)	2 (11%)
Core biopsies performed	89 (92%)	78 (100%)	11 (58%)
Core biopsy gauge			
18 or 19	6 (6%)	4 (5%)	2 (11%)
20 or 21	83 (86%)	74 (95%)	9 (47%)
Core biopsy count	3.0 (2.0-4.0)	3.0 (2.0-4.0)	3.0 (2.5-3.0)
Any specimen cultured	8 (8%)	8 (8%)	0
Skin-to lesion, millimeters	60.5 (45.0-82.5)	65.0 (47.0-83.0)	47.0 (25.0-80.0)
Number of pleural passes*			
One	81 (84%)	66 (85%)	15 (79%)
More than one	14 (14%)	12 (15%)	2 (11%)
Number of fissures crossed			
Zero	89 (92%)	71 (91%)	18 (95%)
One	7 (7%)	7 (9%)	0
ROSE assessment			
Malignant	5 (5%)	5 (6%)	0
Inadequate for immediate malignant diagnosis	2 (2%)	2 (3%)	0
Not utilized	88 (91%)	71 (91%)	17 (89%)
Procedure duration, minutes	24.5 (13.0-36.0)	23.5 (13.0-35.8)	26.5 (14.5-38.0)
Proceduralist assessed successful biopsy	89 (92%)	77 (99%)	12 (63%)

n (%) or median (IQR)

*"Pleural pass" here refers to the number of times a transthoracic needle passed through a visceral pleural membrane, which is not necessarily the same as the number of transthoracic biopsies obtained in a given case, as most cases utilized a coaxial needle which crossed the visceral pleura just once, through which multiple core biopsies or fine needle aspirates were subsequently obtained.

Additional invasive diagnostic and staging procedures (secondary outcome)

Additional biopsy targeting the same nodule targeted by study biopsy was pursued in 31 nondiagnostic cases (13%), detailed in the table below. The subsequent biopsy was diagnostic in most cases (81%). A staging bronchoscopy was pursued after 3 (3%) of transthoracic biopsies; no additional staging procedures were pursued in the bronchoscopy arm.

Table S9. Additional invasive diagnostic and staging procedures.

	NB (n=121)	TTNB (n=113)	Overall (n=234)	Difference (95% CI)
Subsequent procedure	16 (13%)	15 (13%)	31 (13%)	0.1% (-8.8 to 8.7)
Bronchoscopy	2 (2%)	12 (11%)	14 (6%)	9% (2 to 15.9)
Surgical biopsy/resection	10 (8%)	2 (2%)	12 (5%)	6.5% (0.2 to 12.8)
TTNB	4 (3%)	0	4 (2%)	3.3% (0.7 to 7.3)
Liquid biopsy	0	1 (1%)	1 (0.4%)	0.9% (-3.5 to 1.7)
Interval, study biopsy to additional biopsy, d	48.0 (34.8-74.5)	49.0 (10.5-98.5)	48.0 (27.5-88.0)	1 (-90 to 67)
Subsequent procedure was diagnostic	13 (81%)	12 (80%)	25 (81%)	1.2 (-28 to 30)
Additional staging procedure	0	3 (3%)*	3 (1%)	3.0 (-40 to 6.7)

n (%) or median (IQR)
*Staging linear endobronchial ultrasound-facilitated transbronchial needle aspiration in all cases

Confident clinical diagnosis (secondary outcome)

Confident clinical diagnosis was defined as the proportion of study interventions which yielded a confident clinical diagnosis based on pathology results plus 1) linear endobronchial ultrasound-guided transbronchial needle aspiration (EBUS-TBNA) of central lymph nodes and 2) culture data. EBUS-TBNA revealed metastases to mediastinal or hilar nodes in two bronchoscopy cases, both of which also revealed malignancy in nodule biopsies. There were no instances of culture data providing a diagnosis in cases where nodule biopsy pathology was deemed nondiagnostic. Therefore, the proportion of cases with a confident clinical diagnosis was identical to the diagnostic yield: for bronchoscopy, 96/121 (79.3%) compared to 88/113 (77.6%) for transthoracic biopsy, mean difference 1.5 percentage points, 95% CI -9.9 to 12.8.

Author Contributions

Study concept and design: FM, RJL, OBR, KFD, VP, TK, EG, LY, GS, NR

Acquisition of data: RJL, KFD, VP, MCA, BS, LR, SWL, CS, SKA, TCH, MW, KM, GC, JMK, JSK, JJ, PFD, CW, OBR, FM, JDD, SS

Analysis and interpretation of data: RJL, FM, TK, JC, NR

Drafting of the manuscript: RJL, FM, JC, NR

All authors participated in critical revision of the manuscript for important intellectual content and provided final approval to submit this version of the manuscript and have agreed to be accountable for all aspects of the work.

Supplementary References:

1. McWilliams A, Tammemagi MC, Mayo JR, et al. Probability of cancer in pulmonary nodules detected on first screening CT. *N Engl J Med* 2013;369(10):910–919.

2. Herder GJ, Tinteren H van, Golding RP, et al. Clinical prediction model to characterize pulmonary nodules: validation and added value of 18F-fluorodeoxyglucose positron emission tomography. *Chest* 2005;128(4):2490–2496.

3. Thiboutot J, Lee HJ, Silvestri GA, et al. Study Design and Rationale: A Multicenter, Prospective Trial of Electromagnetic Bronchoscopic and Electromagnetic Transthoracic Navigational Approaches for the Biopsy of Peripheral Pulmonary Nodules (ALL IN ONE Trial). *Contemp Clin Trials* 2018;71:88–95.

4. Aboudara M, Roller L, Rickman O, et al. Improved diagnostic yield for lung nodules with digital tomosynthesis-corrected navigational bronchoscopy: Initial experience with a novel adjunct. *Respirology* 2019;

5. Katsis J, Roller L, Aboudara M, et al. Diagnostic Yield of Digital Tomosynthesis-assisted Navigational Bronchoscopy for Indeterminate Lung Nodules. *J Bronchology Interv Pulmonol* 2021;28(4):255–261.

6. Avasarala SK, Roller L, Katsis J, et al. Sight Unseen: Diagnostic Yield and Safety Outcomes of a Novel Multimodality Navigation Bronchoscopy Platform with Real-Time Target Acquisition. *Respiration* 2021;1–8.

7. DiBardino DM, Yarmus LB, Semaan RW. Transthoracic needle biopsy of the lung. *J Thorac Dis* 2015;7(Suppl 4):S304-316.

8. Vachani A, Zhou M, Ghosh S, et al. Complications After Transthoracic Needle Biopsy of Pulmonary Nodules: A Population-Level Retrospective Cohort Analysis. *J Am Coll Radiol* 2022;19(10):1121–1129.

9. Wiener RS, Schwartz LM, Woloshin S, Welch HG. Population-based risk for complications after transthoracic needle lung biopsy of a pulmonary nodule: an analysis of discharge records. *Ann Intern Med* 2011;155(3):137–144.

10. Lentz RJ, Frederick-Dyer K, Planz VB, et al. Navigational Bronchoscopy vs CT Scan-Guided Transthoracic Needle Biopsy for the Diagnosis of Indeterminate Lung Nodules: Protocol and Rationale for the Navigation Endoscopy to Reach Indeterminate Lung Nodules vs Transthoracic Needle Aspiration, a Randomized Controlled Study Multicenter Randomized Trial. *CHEST Pulmonary* [Internet] 2024 [cited 2024 Sep 23];2(3). Available from: https://www.chestpulmonary.org/article/S2949-7892(24)00016-3/fulltext

11. Gould MK, Tang T, Liu I-LA, et al. Recent Trends in the Identification of Incidental Pulmonary Nodules. *Am J Respir Crit Care Med* 2015;192(10):1208–1214.

12. Cai J, Vonder M, Du Y, et al. Who is at risk of lung nodules on low-dose CT in a Western country? A population-based approach. *Eur Respir J* 2024;63(6):2301736.

13. National Lung Screening Trial Research Team, Aberle DR, Adams AM, et al. Reduced lung-cancer mortality with low-dose computed tomographic screening. *N Engl J Med* 2011;365(5):395–409.
